# Changes in Human Langerhans Cells Following Intradermal Injection of Influenza Virus-Like Particle Vaccines

**DOI:** 10.1371/journal.pone.0012410

**Published:** 2010-08-25

**Authors:** Marc Pearton, Sang-Moo Kang, Jae-Min Song, Alexander V. Anstey, Matthew Ivory, Richard W. Compans, James C. Birchall

**Affiliations:** 1 Welsh School of Pharmacy, Cardiff University, Cardiff, United Kingdom; 2 Department of Microbiology and Immunology, Emory University School of Medicine, Atlanta, Georgia, United States of America; 3 Aneurin Bevan Health Board Royal Gwent Hospital, Newport, United Kingdom; New York University, United States of America

## Abstract

There is a significant gap in our fundamental understanding of early morphological and migratory changes in human Langerhans cells (LCs) in response to vaccine stimulation. As the vast majority of LCs studies are conducted in small animal models, substantial interspecies variation in skin architecture and immunity must be considered when extrapolating the results to humans. This study aims to determine whether excised human skin, maintained viable in organ culture, provides a useful human model for measuring and understanding early immune response to intradermally delivered vaccine candidates. Excised human breast skin was maintained viable in air-liquid-interface organ culture. This model was used for the first time to show morphological changes in human LCs stimulated with influenza virus-like particle (VLP) vaccines delivered via intradermal injection. Immunohistochemistry of epidermal sheets and skin sections showed that LCs in VLP treated skin lost their typical dendritic morphology. The cells were more dispersed throughout the epidermis, often in close proximity to the basement membrane, and appeared vertically elongated. Our data provides for increased understanding of the complex morphological, spatial and temporal changes that occur to permit LC migration through the densely packed keratinocytes of the epidermis following exposure to vaccine. Significantly, the data not only supports previous animal data but also provides new and essential evidence of host response to this vaccination strategy in the real human skin environment.

## Introduction

**Figure 1:**
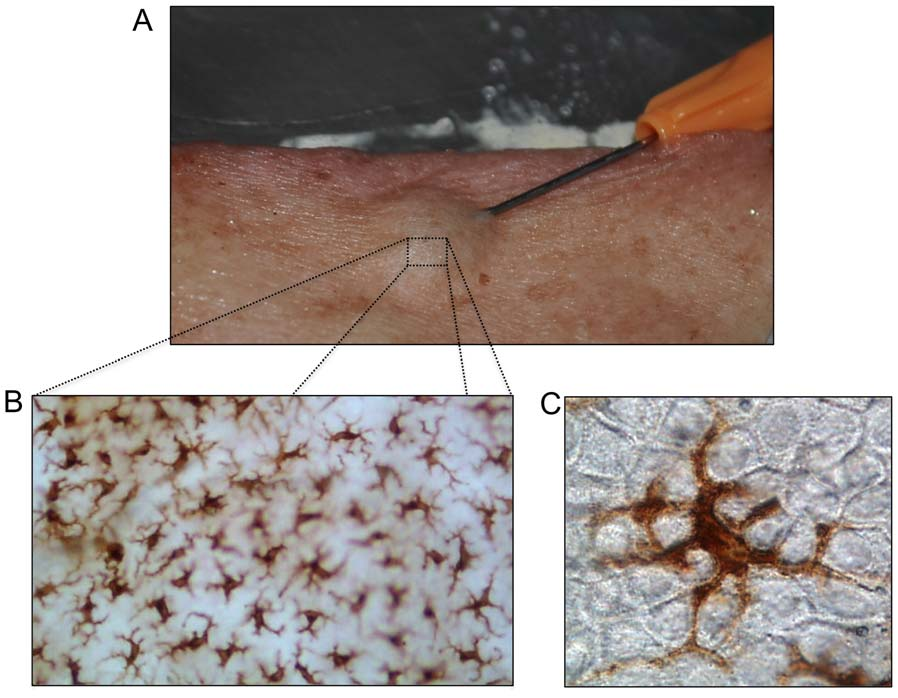
Rationale for intradermal (ID) vaccine delivery. (**A**) ID delivery of H1 VLPs to excised human skin using the Mantoux method. A 26G hypodermic needle was inserted laterally into the skin followed by injection of H1 VLP suspension (10µg VLP). A characteristic wheal, typical of ID injection, is clearly visible. (**B**) IHC stained (CD207) human epidermal sheet showing the extensive network characteristic of LCs. (**C**) A single LC displaying the typical dendritic cell nature of this cell type. Dendrites emanating from the LC body can clearly be seen infiltrating between individual keratinocytes.

Intradermal (ID) delivery of vaccines has been shown to induce protective immunity against many diseases, including hepatitis B [1], [2], rabies [3], tuberculosis [4], measles [5], polio [6] and influenza [7], [8]. ID vaccination aims to exploit the abundance of antigen presenting cells (APCs) found within the skin; that is dermal dendritic cells (DDCs) in the dermis and LCs in the epidermis. Both these cell types have the ability to uptake, process and present both self- and foreign antigens to naïve T-cells [9] following migration to the lymphatics, and therefore have the potential to elicit an adaptive immune response [10]. Administration of vaccines by the ID route therefore aims to target these cell types (Figure 1) to provide a more proficient immune response, potentially at reduced dose [11]. Whilst we acknowledge the potential importance of both of these cell types in generating an immune response, in this study we will focus solely on epidermal LCs as the most accessible and easily monitored dendritic cell (DC) subtype.

LCs comprise approximately 3–5% of the nucleated cells in the epidermis [12] and possess a typical DC morphology [13], with numerous cytoplasmic processes penetrating the intracellular regions between keratinocytes (Figure 1C). LCs are found in sufficient numbers to form a complete network across the entire skin surface with the dendrites from individual LCs extending out from the cell body in parallel to the basement membrane. LCs are generally observed towards the centre of the epidermis (stratum spinosum), surrounded on all sides by keratinocytes. The migratory nature of antigen-stimulated LCs has been well characterized in rodent models, providing insight into the behavior of this cell type [14], [15]. LC activation and migration has been shown to depend on differing levels of cytokines and chemokines produced by the LCs themselves and the surrounding keratinocytes. Principle cytokines in this respect are interleukin-1β (IL-1β), tumor necrosis factor-α (TNF-α), and interlukin-18 (IL-18) [16]. Upon activation, LCs negotiate a path through the surrounding keratinocytes and migrate across the epidermal/dermal junction. During transit, antigen-activated LCs mature, resulting in a reduced ability to process subsequently encountered antigen and an increase in immuno-stimulatory capabilities [17]. The ultimate destination of actively migrating LCs is the paracortical regions of lymphatic nodes where presentation of peripherally acquired antigen to naïve T-cells occurs [13].

Although there are some reports investigating the immunological role and migratory nature of human LCs [18], [19], the vast majority of LCs studies have been conducted in small animal models, principally the mouse [20]–[22]. As a corollary, substantial interspecies variation in skin architecture and immune response must be taken into consideration when extrapolating these data to the situation in humans [23]. In particular, despite numerous clinical studies reporting ID administration with influenza vaccines [24]–[30], there is a significant gap in our understanding of the initial events that occur in human skin. Since LCs reside in the epidermis, their accessibility provides a unique opportunity to study a subset of APC in their native environment. This study utilizes a previously established *ex vivo* human skin organ culture system [31] to examine the early morphological and behavioral changes of human LCs in response to a promising vaccine candidate; influenza virus-like particles (VLPs) [32]–[35]. Using this model of real human skin, we aim to provide unique mechanistic information relating to the poorly defined sequence of events occurring immediately following LCs contact with vaccine antigens that lead to trafficking of the LCs to the lymphatic system. While this kind of study of human LCs would be difficult in human patients, the use of cultured human skin explants provides a unique opportunity to monitor these events in a controlled environment. An increased understanding of antigen-induced activation and migration of LCs in human skin tissue will serve to complement animal and human ID vaccine studies, providing a meaningful data set to inform pre-clinical and clinical vaccine development.

## Results

### Changes in LCs numbers following ID injection of H1 VLP vaccines

**Figure 2:**
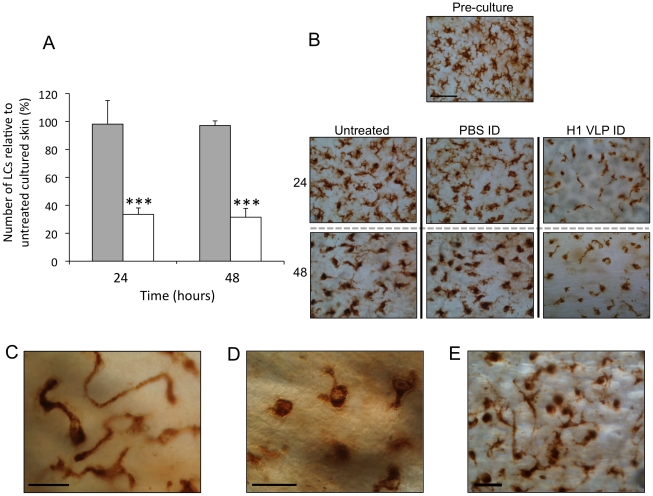
Changes in LC numbers and morphology in epidermal sheets. Human epidermal sheets were isolated from untreated cultured skin and cultured skin treated by ID injection of PBS (control) or H1 VLPs. (**A**) Epidermal sheets were stained for CD207 and positively stained cells were counted. Data presented as the percentage of CD207+ve cells (LCs) relative to cell numbers in untreated skin at each timepoint. Skin treated by ID delivery of PBS (grey bars); Skin treated by ID delivery of H1 VLPs (white bars); data presented as mean ± SD (n = 4), significance was determined relative to untreated skin at each timepoint (***p<0.001). (**B**) Representative images of LCs (CD207+ve cells) taken from each time point and treatment (bar = 20µm). (**C**) LCs displaying a “hyper-dendritic “cell morphology. (**D**) LCs displaying “rounded” cell morphology. (**E**) Both hyper-dendritic and rounded LCs observed in the same optical plane of the epidermis (bar = 10µm all cases).

On excision and subsequent culture of human skin, LCs will inevitably begin to migrate from the epidermis due to physical and chemical changes in the tissue resulting in the propagation of migratory signals [15], [31], [36]. This non-antigen mediated depletion in LCs numbers in epidermal sheets is apparent in untreated human skin samples (Figure 2B) and as such represents the baseline against which LCs changes in skin samples in response to injected vaccine can be measured.

Following ID injection of influenza H1 VLP vaccine, a more pronounced effect on the number of LCs in epidermal sheets was observed. Epidermal membranes prepared 24 hours after injection with HI VLPs were observed to contain significantly (P<0.001) lower numbers of LCs compared to untreated skin samples at the same time point (Figure 2A). The more marked reduction in LCs numbers was also obvious 48 hours after treatment. The number of LCs in epidermal sheets taken from skin samples injected with PBS alone did not differ from untreated skin samples. Representative images of epidermal sheets are displayed in Figure 2B. The images serve to reflect the significant reduction in LCs numbers observed when skin is injected with H1 VLPs compared with blank skin and skin injected with PBS alone. These data provide clear evidence that LCs in excised human skin migrate from a given optical plane of the epidermis in greater numbers following ID injection of H1 VLPs compared to control skin. We now aim to further explore the nature of LC response to VLP vaccine in human skin.

### Changes in LCs morphology following ID injection with H1 VLP vaccines

During the microscopic analysis of cell numbers in epidermal sheets, on closer inspection we observed that LCs underwent vivid changes in cell morphology, particularly following treatment with VLPs. Populations of LCs formed single, extremely long dendrites (Figure 2C), so termed a “hyper-dendritic” morphology. Additionally, a considerable proportion of LCs did not have any surface protrusions at all (Figure 2D), these were described as having a “rounded” morphology. Frequently, both hyper-dendritic and rounded LCs were observed in close proximity (Figure 2E), indicating that individual cells within defined regions, were responding either differently or at different rates from one another in response to administered vaccine. It was noted that these morphological changes were greatly more pronounced in skin treated with H1 VLPs with both hyper dendritic and rounded LCs constituting a larger proportion of the overall LC population compared to untreated skin.

### LCs dendrite morphology

**Figure 3:**
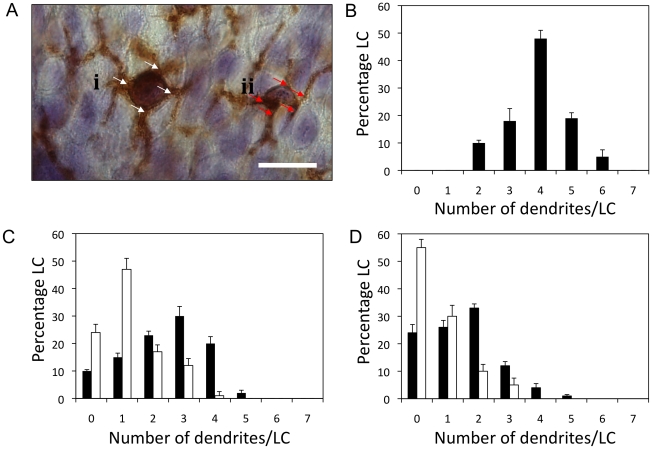
Number of dendrites per LC in epidermal sheets. (**A**) Microscopic examination revealed each LC to have a distinct cell body from which dendrites protruded (bar = 10µm). Two individual LCs designated (i) and (ii) with 4 and 5 dendrites per cell, indicated by the white and red arrows, respectively. Percentage frequency of dendrites per LC in (**B**) pre-cultured epidermal sheets and post-cultured epidermal sheets at 24 (**C**) and 48 (**D**) hours; untreated skin (black bars), skin treated with H1 VLPs delivered by ID injection (white bars). Data presented as mean ± SEM (n = 8).

In an attempt to quantify the aforementioned morphological changes observed in LCs as a consequence of exposure to H1 VLPs, the numbers of dendrites emanating from individual LCs were calculated. The typical appearance of LCs found in epidermal sheets isolated from untreated *ex vivo* human skin approximately 1.5 hours post surgery (the time at which *ex vivo* skin organ culture begins) is shown as an example in Figure 3A. In this image one of the LCs (i) has a total of four dendrites protruding from the cell body (white arrows); while the other (ii) has a total of five dendrites (red arrows). When dendrite numbers per LC were calculated in untreated excised human skin in this way, it was observed that a ‘typical’ LC had on average four dendrites/cell (mean = 3.8; median = 4). The number of dendrites/cell assumes a normal distribution around this mean value with some LCs having as many as six dendrites and some as few as two (Figure 3B).

The dendritic appearance of LCs in untreated *ex vivo* human skin changed as the cells responded to the culture environment (Figure 3C and 3D). A general trend toward less dendrites/cell was observed following 24 hours tissue culture (mean = 2.466; median = 3), providing a baseline value for observations of treated samples at that time point. Skin treated with H1 VLPs administered by ID injection showed a greater reduction in dendrite numbers at 24 hours (mean = 1.252; median = 1) compared to untreated skin (Figure 3C). Whilst untreated skin samples showed further reductions in dendrite number/LC at 48 hours, the samples exposed to H1 VLPs were again shown to contain cells with even fewer dendrites (Figure 3D). At this later time point, many of the LCs in untreated blank skin still maintained a degree of dendritic morphology (mean = 1.490; median 1.5); however cells displaying dendritic morphology were much less apparent in skin treated with H1 VLP via ID injection (mean = 0.650; median = 0).

### Area of LCs in epidermal sheets

**Figure 4:**
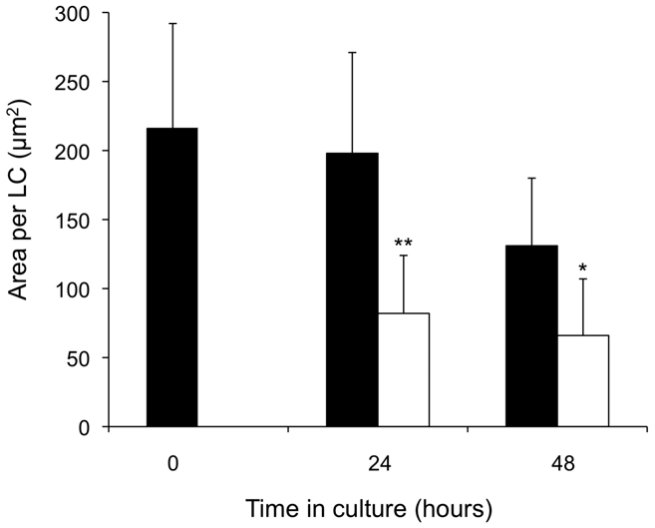
Area of individual LCs in epidermal sheets. The mean individual LC area (n = 100) was determined using ImageJ software following culturing of blank skin (black bars) and skin treated with H1 VLP delivered by ID injection (white bars). Data presented as mean ± SD (n = 4); significance was determined relative to blank skin at corresponding time points (*p<0.05).

*En face* images captured at high magnification (×100) were used to determine the mean area occupied by individual LCs within epidermal sheets (Figure 4). In blank, untreated skin samples no significant difference was observed in mean LC area between skin samples at 1.5 hours post-surgery and at 24 hours in organ culture. However, at 48 hours there was a significant reduction in individual LC area (p<0.05) as the skin responds to prolonged organ culture. At 24 hours the mean LC area in samples treated with H1 VLP by ID injection was significantly reduced (p<0.05) compared to untreated, blank skin (Figure 4). Interestingly, and contrary to untreated samples, mean LC area in VLP treated samples remained virtually unchanged between 24 and 48 hours, possibly suggesting that the immune stimulatory effects of H1 VLP vaccines occur in the first 24 hours after administration.

### Area, morphology and distribution of LCs in sections

**Figure 5:**
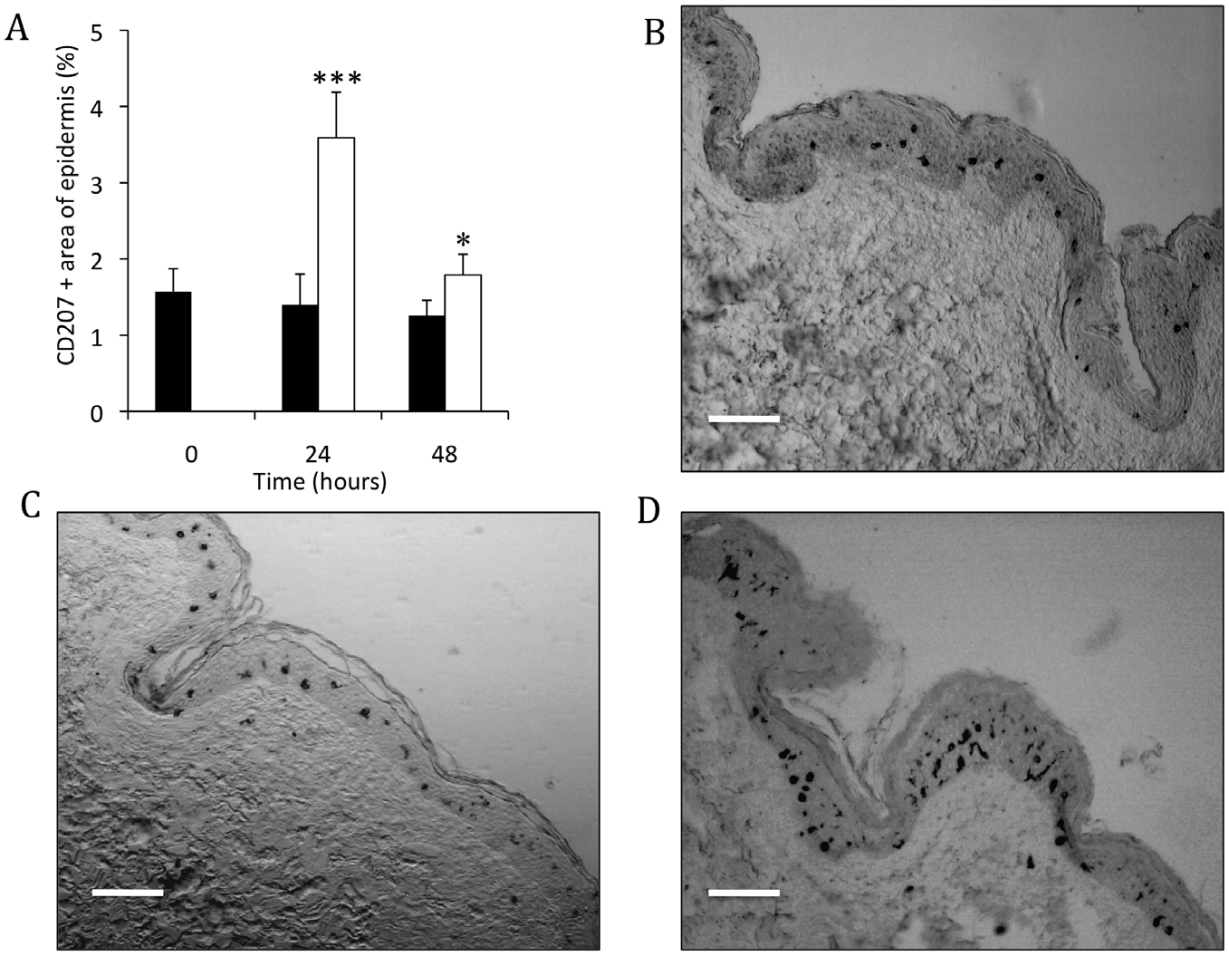
Relative area and spatial distribution of LCs in histological sections. (**A**) The relative area of CD207+ve cells as a percentage of total epidermal area in skin sections was determined using ImageJ software. Data presented as mean ± SD (n = 4) significance was determined relative to blank skin at each corresponding time point (*p<0.05, **p<0.01, ***p<0.001). Skin sections showing spatial distribution of LCs in histological skin sections: untreated skin at 0 hours (**B**), untreated skin at 24 hours (**C**) and skin treated with H1 VLPs delivered by ID injection at 24 hours (**D**). Bar = 50µm in all cases.

Following assessments of LC morphology in a single horizontal plane, i.e. in epidermal sheets, further experiments were conducted to investigate changes in LC morphology and distribution throughout the vertical skin strata. In the first analysis the total area occupied by LCs as a percentage of total epidermal area was determined following image analysis of skin sections. In transverse sections of untreated skin the area occupied by LCs remains relatively consistent over the culture period (0–48 hours; Figure 5A). The sections reveal LCs to be typically rounded and well dispersed throughout the epidermis, with no noticeable overlapping of cells (Figure 5B,C). In contrast, sections of skin following ID injection of H1 VLP vaccines showed significant changes in total area occupied by LCs (P<0.01), LC morphology and LC distribution pattern. LC area in skin sections prepared 24 hours post-injection of VLPs was approximately doubled (Figure 5A). The spatial distribution of LCs in these samples was also noticeably different to blank skin at the same time points. For example, Figure 5C and D show representative sections of untreated skin and H1 VLP treated skin respectively at 24 hours. The LCs in the H1 VLP treated samples (Figure 5D) appeared to be more dispersed though the epidermis and less uniform in distance from one another. Frequently, LCs were observed to be distributed on top of each other, whereby one LC was typically located toward the centre of the epidermis with another in close proximity to the basement membrane (Figure 5D). This distribution pattern was not observed in blank skin (Figure 5C). At 48 hours the total area occupied by LCs in skin sections had returned to approximately the same value pre-culture, with no significant difference observed between blank and treated samples (Figure 5A).

**Figure 6:**
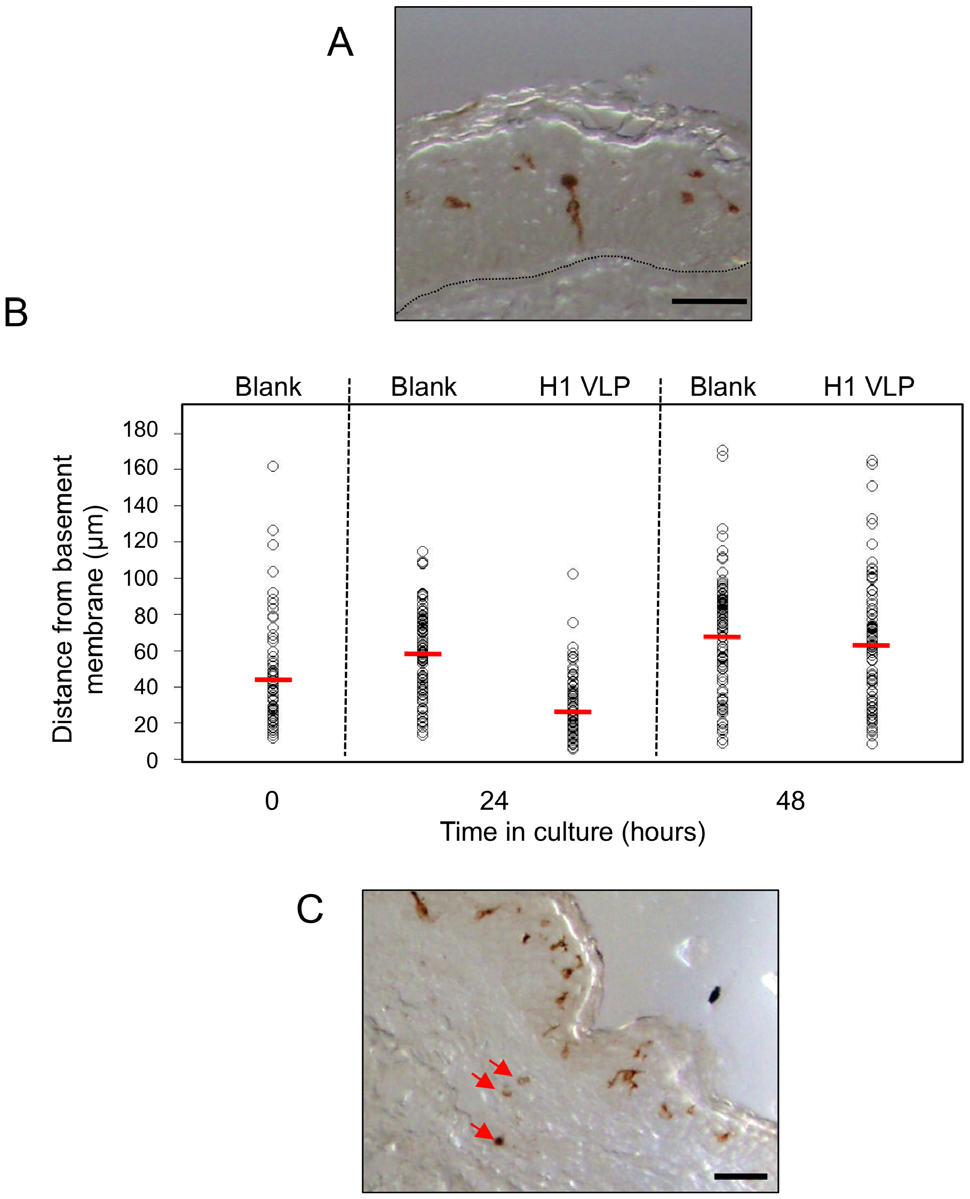
LCs localization to epidermal basement membrane. (**A**) IHC stained (CD207) histological section showing a single LC extending an exploratory dendrite toward the basement membrane; dashed line indicates the epidermal/dermal interface (bar = 20 µm). (**B**) The mean distance of LCs from the basement membrane in untreated and VLP-treated skin samples. (**C**) Representative examples of CD207+ve cells located in the skin dermis (red arrows) 24 hours after ID injection of H1 VLPs (bar 50 µm).

The transverse sections of H1 VLP treated skin samples in Figure 5D show that LCs appeared to be vertically elongated following vaccine administration. A clear example of a single LC displaying this elongated morphology is shown in Figure 6A. A more detailed analysis of individual and mean LC distance from the epidermal membrane as observed in histological sections is presented in Figure 6B. The mean LC distance from the epidermal membrane is markedly reduced following ID injection of H1 VLPs, this being particularly noticeable at the 24 hours time point.

Figure 5D previously showed the presence of CD207+ve cells located in the dermis. Figure 6C provides an image of another section of skin injected with H1 VLPs 24 hours earlier. Whilst the majority of CD207+ve cells, many of which are elongated, appear in the epidermis, a small number of cells can also be seen beneath the epidermal membrane (see arrows) in this section. CD207+ve cells were very rarely observed in the dermis in blank skin samples. Whilst in this paper we focus on LC behavior in human epidermis we feel this additional observation of CD207+ve cells in the dermis warrants a further human skin study aimed at tracking antigen-associated changes to immune-responsive cells from epidermis to dermis to lymph vessel.

## Discussion

Recent studies have demonstrated that influenza VLPs, as a particulate antigen mimicking native virions structurally and morphologically, confer 100% protection in mice even after a single dose vaccination [32], [37]. We have also observed that the H1 VLPs used in this study were highly immunogenic and conferred protection after a single dose of ID injection in an animal model (data not shown). More importantly, influenza VLPs showed an equivalent or better efficacy compared to traditional influenza vaccines in a recent clinical trial [38] indicating that influenza VLPs can be a clinically important vaccine candidate. For the first time, the current study aims to demonstrate that influenza VLPs, as a relevant vaccine candidate, delivered via ID injection, have a stimulatory effect on live, functional human DCs in the biologically relevant environment of viable human skin.

DCs are highly proficient at uptaking, processing and presenting antigenic material from the surrounding milieu to naive, antigen specific T-cells within the nodes of the draining lymphatic system [14]. LCs are peripheral DCs that therefore have to migrate from the site of antigen uptake to present the antigen to T-cells. The focus of this study was to observe and monitor LCs behavior in viably maintained excised human skin following ID injection of influenza H1 VLP vaccines. The data presented in this paper was obtained by injecting two donor skin samples with a single batch of H1 VLPs. The morphological and spatial changes we observe however generally reflect other studies we have performed using different delivery methods [39] and VLP strains (manuscript in preparation).

Previous studies have demonstrated the properties of LCs in skin organ culture [31], [39], [40], [41] and their responses to changes in pH, oxygen levels, damage generated by excision, excess hydration and accumulation of waste products. Despite these changes in LCs caused by changes in their environment, our work suggests that a 48 hour window exists in which to investigate the early stages of LC activation and migration in response to antigen exposure. Freshly excised human skin was maintained viable in organ culture following ID injection of H1 VLP vaccine. Untreated skin and skin injected with PBS alone were also cultured as controls. Following skin incubation at air-liquid interface for 24 or 48 hours epidermal sheets were generated and stained for CD207 to identify and quantify LCs populations. The number of LCs observed in epidermal sheets depleted significantly more rapidly when skin had been treated with H1 VLPs delivered via ID injection, compared to blank skin and PBS control, confirming LC migration in response to ID vaccine delivery. The fact that a 10µl injection of PBS alone failed to reduce LC numbers any more than untreated controls, and that VLP administration via minimally invasive microneedles has also been shown to reduce LC numbers [39], suggests that the observed changes in LC response are due to antigenic stimulation rather than any inflammation or trauma caused by the administration procedure. This study reports for the first time the observation that vaccination with VLPs via ID injection can stimulate the migration of LCs from the human skin epidermis.

In order for antigen-stimulated LCs to migrate, a number of morphological changes need to occur. In intact normal skin viewed *en face*, LCs spread over the body surface forming a network with many characteristic dendritic protrusions, as observed in pre-cultured skin samples. Migration of the LC to the lymphatic vessels in the dermis requires the cell to negotiate a tortuous path through the tightly packed keratinocytes resident in the epidermis. In the unstimulated state the highly dendritic morphology of the LC would restrict the movement between epidermal keratinocytes. It is proposed that LCs displaying highly dendritic morphologies are indicative of stationary, non-motile cells. In order to aid physical movement through the epidermis, LCs must therefore retract their dendrites upon antigen stimulation and this should be apparent when viewed *en face*. Results from this study showed that when skin was treated with H1 VLP vaccines a more pronounced reduction in dendrites per LC was observed, when compared with untreated skin at 24 and 48 hours. Indeed, direct *en face* observation of epidermal sheets at these time points revealed that H1 VLP treated samples contained high proportions of LCs with retracted dendrites, thus displaying a “rounded” morphology. Also present, in considerable numbers in these samples were LCs with a single “hyper-dendritic” protrusion. According to a study performed by Swetman et al., 2002, using an *in vitro* DC model, in order for DCs to move, an initial point of attachment is made to the extra-cellular matrix, and then the cell body moves away from this point leaving behind a long, filamentous dendrite, in a process known to be dependent on Rho GTPase [42]. With regard to the present study, this suggests that LCs displaying highly elongated hyper-dendritic morphologies were likely to be actively in the process of migrating through the epidermis toward the basement membrane in response to antigen; similar morphological and spatial observations being observed in the mouse following LCs stimulation [22].

Image analysis of the CD207 stained epidermal sheets revealed that as the number of dendrites reduced, during retraction, the individual cell area also reduced. In contrast, when skin samples were examined in histological sections, at equivalent time points, there was a noticeable increase in total LCs area at 24 hours. Rather than contradicting the changes in LC number and individual LC area seen in epidermal sheets, this can be rationalised in light of LC migration events. In order to migrate, dendrites have to be retracted, resulting in a decrease in individual cell area when viewed *en face*. Subsequent movement of the cell towards the basement membrane results in a visible reduction in LC *number* in the optically narrow horizontal plane of an epidermal sheet yet an increase in total cell *area* in the vertical plane of an epidermal section. Indeed, results from this study showed that 24 hours after VLP vaccine treatment individual LC area was halved when viewed *en face* yet total cell area was doubled in transverse section, strongly supporting the hypothesis that morphological changes in LCs are induced differentially through migration upon antigenic stimulation. That is, prior to migration LCs are elongated horizontally, with respect to the skin surface, but upon migration become elongated vertically, with respect to the skin surface. Histological sections of skin prepared 24 hours following treatment with H1 VLPs showed that the LCs were much more dispersed throughout the epidermis, with many cells occupying positions proximal to the basement membrane. In these samples, cells were frequently observed vertically elongated with respect to the skin surface, suggesting downward movement of the cell toward the basement membrane. Indeed, there is also evidence that LCs have migrated across the epidermal membrane into the dermis towards the lymphatic drainage system. The area occupied by remaining epidermal LCs, in histological sections, returned to approximately pre-culture values at 48 hours, which likely reflect a population of LCs that for either unknown specific biological reasons or insufficient or inappropriate stimulation fail to migrate. This has been observed and documented elsewhere where subsequent to antigen stimulation and initial migration a population of LCs remained and failed to migrate even following a second antigen application [43].

**Figure 7:**
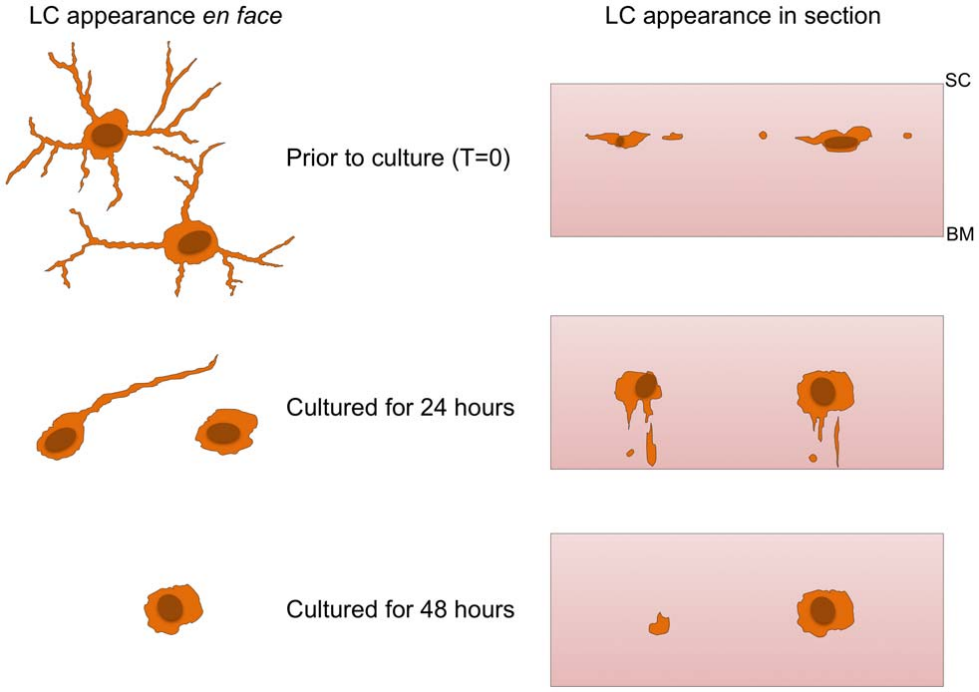
Schematic depicting the proposed sequence of events leading to LC migration from skin epidermis. In normal skin LCs display typical DC morphology, i.e. contain many dendritic protrusions. Upon activation LCs detach from surrounding keratinocytes and retract dendrites assuming a “rounded” morphology. A point of attachment is made from which the LC subsequently moves, potentially in a chemotactic manner, toward the basement membrane. Cells elongate to enable downward vertical movement.

A schematic hypothesis summarizing the observed migratory processes is shown in Figure 7. It is proposed that in response to antigen-induced stimulation LCs begin to retract their dendrites from the horizontal plane and take on a rounded appearance. This provides a point of focus from which the LCs can begin to negotiate a path down and between the tightly packed keratinocytes immediately below. From this rounded state, the LC extrudes a single, exploratory dendrite in between the keratinoctyes, toward the basement membrane. As the exploratory dendrite forges a path between keratinocytes, the LC becomes elongated, extending into the hyper-dendritic morphology described previously. As the antigen stimulated LC approaches the basement membrane the cell once again “rounds up”, before traversing the membrane and continuing into the dermis.

In summary, ID injection of H1 VLP vaccines into *ex vivo* human skin explants induced a marked biological response in endogenous LCs. It appeared as if human LCs followed a predetermined sequence of morphological changes in response to the delivered vaccine, which ultimately ended in significant numbers of cells migrating through and out of the epidermis. Considerable knowledge regarding the activation and migratory nature of LCs has previously been gained from the use of animal models. However, it is critically important that morphological changes and migration of LCs in response to vaccines are confirmed in the human environment, as demonstrated for the first time in this study for VLP vaccines. Monitoring cellular changes in excised human skin can provide new functional and mechanistic insight into the early human responses to vaccines, providing a powerful tool in developing effective future vaccines.

## Materials and Methods

### Ethics Statement

Human skin samples were obtained from female patients undergoing mastectomy or breast reduction surgery under informed written patient consent and local ethical committee approval (South East Wales Research Ethics Committees Panel C, Reference: 08/WSE03/55).

### Materials

Dulbecco's modified Eagle medium (DMEM), fetal calf serum (FCS) and penicillin/streptomycin were purchased from Invitrogen (Paisley, UK); all other items were purchased from Fisher Scientific (Loughborough, UK) unless otherwise stated.

### Collection, transport and processing of human skin

Excised human skin was transported from surgery to the laboratory in culture media composed of 94% DMEM, 5% FCS and 1% penicillin/streptomycin (each at 100 IU mL^−1^) at 4°C. Skin was prepared for treatment by removal of subcutaneous fat tissue by blunt dissection before being pinned, dermis side down, onto a cork dissection board. Treated regions and control untreated regions of skin were excised, each approximately 0.5×0.5 cm, and cultured at the air-liquid interface at 37°C and 5% CO_2_ [31], [44]. Data presented in this study was obtained from numerous replicate samples performed in skin taken from two independent patient donors.

### ID injection of H1 VLP vaccines

Recombinant baculoviruses (BVs) expressing hemagglutinin (HA) and matrix (M1) derived from A/PR/8/34 (H1N1) and influenza VLPs containing HA and M1 were prepared as described [45]. To produce influenza VLP vaccine, Sf9 insect cells were co-infected with recombinant BVs expressing HA and M1. Influenza VLPs in the culture supernatants were purified by using discontinuous sucrose gradient layers, and characterized by Western blot and hemagglutination activity analysis [37], [45]. The content of HA was approximately 10% of total proteins of influenza VLPs, which is similar to that of a previous report [37], [45]. H1 VLPs (10µl of a 1mg/ml solution in PBS) were injected into the dermal compartment of excised human skin. A clear wheal following injection signaled correct positioning of the needle for ID injection (Figure 1) [46]. Control samples received an ID injection of 10µl of sterile PBS.

### Monitoring changes in Langerhans cell numbers, morphology and area in epidermal sheets

Cultured skin samples were incubated in 3.8% w/v ammonium thiocyanate (Sigma-Aldrich chemical company, Poole, UK) in PBS for 20–40 min. Epidermis was peeled away from the dermis with forceps under a dissection microscope. Epidermal sheets were fixed in pre-cooled acetone at −20°C for 20 min followed by rinsing in PBS. Epidermal sheets were transferred into 1.5ml centrifuge tubes and incubated in 0.03% v/v H_2_O_2_ for 5 min at room temperature. Peroxide solution was removed and 1 ml of PBS/Tween (0.01M PBS; 0.05% Tween-20 adjusted to pH 7.4) was added for 3 min. This was removed and replaced a total of 3 times. The primary CD207 (Langerin) mouse monoclonal antibody (12D6, Abcam, Cambridge, UK) was diluted 1/100 and incubated at room temperature for 1 hour. Sheets were rinsed in three changes of 1 ml PBS/Tween, each for 3 min. Detection of primary antibody was achieved with an EnVision+ system-HRP (DAB) kit (Dako UK Ltd, Cambridgeshire, UK) used according to manufacturers' specifications. Stained sheets were spread out on slides and visualized by light microscopy with representative images captured digitally (Olympus, Watford, UK).

### Image analysis of LC numbers, dendrite morphology and area in epidermal sheets

Image analysis was performed with ImageJ software (available at http://rsb.info.nih.gov/ij; developed by Wayne Rasband, National Institutes of Health, Bethesda, MD). To determine cell number, immunohistochemically stained epidermal sheets were viewed at ×20 magnification, corresponding to a field of view of 360 by 450 µm. Random images were captured from which the cell number was counted and averaged. Area measurements and dendrite morphology were determined from individual LCs (n = 100 for each treatment) at either ×40 or ×100 magnification from random fields of view.

### Monitoring changes in distribution of LCs in histological sections

Cultured skin samples were rinsed in PBS before fixation in formalin for 24 hours. Samples were dehydrated in an ethanol gradient and cleared in chloroform prior to incubation in two changes of molten paraffin (56°C) before embedding in fresh paraffin. Histological sections of 4µm were generated (Leica 2125 RT microtome, Milton Keynes, UK) and captured onto Superfrost® Plus slides. Selected slides were rehydrated through an ethanol gradient and subject to heat mediated antigen retrieval in Tris-EDTA Buffer (10mM Tris base; 1mM EDTA; 0.05% v/v Tween 20, in 1L _dd_H_2_O adjusted to pH 9.0) at approximately 95°C for 40 min; following which the slides were maintained in antigen retrieval buffer but removed from heat and allowed to cool for approximately 20 min, at room temperature. Slides were washed in two changes of PBS-Tween before incubation in 0.03% H_2_O_2_ for 5 min at room temperature. Detection and visualization of CD207 was as for epidermal sheets.

### Determination of area of LCs in sections and distribution in relation to basement membrane

The percentage area occupied by LCs (CD207+ cells) in IHC stained sections was determined relative to the total epidermal area using ImageJ software.

### Statistical analysis

Statistical tests comprised one-way analysis of variance (ANOVA) followed by Tukey's *post hoc* analysis performed using MINITAB 13 software (Minitab Ltd., Coventry, UK).
