# Correction: Changes in Human Langerhans Cells Following Intradermal Injection of Influenza Virus-Like Particle Vaccines

**DOI:** 10.1371/annotation/00a3b22e-36a9-4d51-89e5-1e6561e7a1e9

**Published:** 2010-11-03

**Authors:** Marc Pearton, Sang-Moo Kang, Jae-Min Song, Alexander V. Anstey, Matthew Ivory, Richard W. Compans, James C. Birchall

The following information was missing from the Competing Interests section: SMK, RWC, and Emory University have equity interests in Zetra Biologicals, which is developing virus-like particle technology under license from Emory University. The authors state that this does not alter their adherence to all the PLoS ONE policies on sharing data and materials.

